# The influence of parasitism by *Trypanosoma cruzi* in the hematological parameters of the white ear opossum (*Didelphis albiventris*) from Campo Grande, Mato Grosso do Sul, Brazil

**DOI:** 10.1016/j.ijppaw.2019.03.015

**Published:** 2019-03-24

**Authors:** Wesley Arruda Gimenes Nantes, Wanessa Teixeira Gomes Barreto, Filipe Martins Santos, Gabriel Carvalho de Macedo, Andreza Castro Rucco, William de Oliveira Assis, Grasiela Edith de Oliveira Porfírio, Gisele Braziliano de Andrade, Ana Maria Jansen, Heitor Miraglia Herrera

**Affiliations:** a Programa de Pós-Graduação em Ciências Ambientais e Sustentabilidade Agropecuária, Universidade Católica Dom Bosco, Tamandaré Avenue, 6000. Jardim Seminário, Cep 79117-900, Campo Grande, Mato Grosso do Sul, Brazil; b Programa de Pós-Graduação em Ecologia e Conservação, Universidade Federal de Mato Grosso do Sul, Costa e Silva Avenue, Cep 79070-900, Campo Grande, Mato Grosso do Sul, Brazil; c Programa de Pós-Graduação em Biotecnologia, Universidade Católica Dom Bosco, Tamandaré Avenue, 6000. Jardim Seminário, Cep 79117-900, Campo Grande, Mato Grosso do Sul, Brazil; d Laboratório de Biologia de Tripanosomatídeos, Instituto Oswaldo Cruz, Fundação Oswaldo Cruz, Brazil Avenue, 4365, Manguinhos, Rio de Janeiro, Rio de Janeiro, Brazil

**Keywords:** *Trypanosoma*, Marsupial, Health, Urban fragment

## Abstract

Considered ecologically generalist, *Didelphis albiventris* is reported as reservoir for different species of parasites, especially *Trypanosoma cruzi*. However, the knowledge about the influence of *T. cruzi* on hematological parameters of free-living opossum remains scarce. The present study aimed to evaluate the influence of *T. cruzi* on hematological parameters of white-ear opossums (*D. albiventris*) from Campo Grande, Mato Grosso do Sul, Brazil. The blood samples and biometric data were collected from 40 opossums captured by Tomahawk and Sherman traps in six urban forest fragments located in the city. The health of these animals was inferred, mainly, by means of blood parameters (PCV, RBC, WBC, MCV and WBC differential). Molecular detection of *T. cruzi* infection was performed by nested polymerase chain reaction (nPCR), using 18S and 24Sα rDNA region as target. Paired-t-test and simple linear regression were used for statistical analysis. No significant difference was observed between the averages of hematological variables in relation to gender and body condition. The molecular diagnosis showed that 32.5% (13/40) of the opossums were infected by *T. cruzi*, which presented lymphocytosis (3.4 ± 1.5) and eosinophilia (0.09 ± 0.13). Path analysis showed that *T. cruzi* infection resulted in increased numbers of lymphocytes and indirectly decreased the body condition of opossums. Moreover *T. cruzi* infection resulted in a direct effect on decrease of MCV. Overall, our results suggest that *T. cruzi* infection may represent a risk to health of opossums since the lymphocytosis may cause a secondary damage on body condition of infected animals.

## Highlights

• *Trypanosoma cruzi* infection may pose a risk to health of opossums.
• Lymphocytosis may cause a secondary damage on body condition of infected animals.
• *D. albiventris* is an important host of *T. cruzi* in the Campo Grande city.

## Graphical abstract

**Figure:**
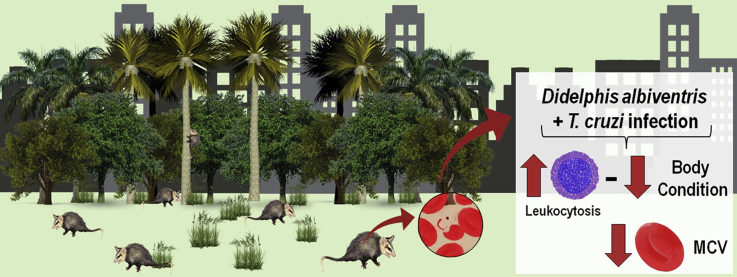

## 1 Introduction

The genus *Didelphis* is widely distributed throughout the Americas (Austad, 1988), from Canada to Argentina. Commonly known as white-ear opossum, *Didelphis albiventris* (Lund, 1840), is found in the Neotropical Region, inhabiting savannahs, gallery forests and damp forests (Emmons; Feer, 1997). In Mato Grosso do Sul it occurs in the Cerrado, Atlantic Forest, Pantanal and in the transition zone between the Chaco and Dry Amazonian Forest (Cáceres et al., 2008; Tomas et al., 2017).

Considered ecologically generalist, this marsupial is frequently in contact with human populations due to their adaptability to anthropized areas and climate changes (Olifiers et al., 2005; Cruz-Salazar et al., 2014). Moreover, *D. albiventris* is commonly reported as reservoir for different species of parasites (Zanette et al., 2008; Humberg et al., 2012; Zabott et al., 2017; Tarragona et al., 2018; Jansen et al., 2018).

The parasitism has different gradients of metabolic interdependence over time, and it is usually considered a negative interaction (Araújo et al., 2003; Lenzi and Vannier-Santos, 2005). Depending on intrinsic factors related to (i) the parasite (quantity of inoculum, different strains with different degrees of virulence and/or pathogenicity and co-infections), (ii) the host (nutritional status, age, sex, reproductive condition and breed), and (iii) environment (food scarcity, severe climatic conditions, fragmentation/decrease of the original habitat and global warming), parasitism can weaken organic conditions and change homeostasis of parasitized individuals (Poulin and Combes, 1999), resulting in a clinical manifestation.

The ancestors of marsupials are reported as the earliest hosts of *T. cruzi* (Roque; Jansen, 2014), and *D. albiventris* is known as one of the most important reservoir hosts for *T. cruzi* in the urban, peri-urban and natural environment (Fernandes et al., 1991; Tenório et al., 2014; Jansen et al., 2015, 2017; Orozco et al., 2016; Roman et al., 2018). However, the knowledge about the influence of *T. cruzi* on hematological parameters of free-living opossum remains scarce. Since studies showed harmful effects of *T. cruzi* infection on free-living mammals (Alves et al., 2011; Olifiers et al., 2015; Carnevali et al., 2017; Santos et al., 2018), the present study aimed to evaluate the influence of *T. cruzi* infection on hematological parameters of white-ear opossums (*D. albiventris*) from Campo Grande, Mato Grosso do Sul, Brazil.

## 2 Material and methods

### 2.1 Capture

This study was carried out between May and December 2017, in six urban forest fragments located in the city of Campo Grande, Mato Grosso do Sul, Brazil. Individuals of *D. albiventris* were captured by Tomahawk and Sherman traps arranged in transects of 200 m with 20 traps each and spacing of 10 m between them. The traps were checked early in the morning, baited and reassembled during the same period. Traps were baited with banana, peanut butter, sardines and oats.

All field procedures were conducted in accordance with a license granted by the Instituto Chico Mendes de Conservação da Biodiversidade (license number 56912-2) and Imasul (license number 05/2017, process Nº61/405959/2016). The present study was approved by the Ethics Committee for Animal Use of Universidade Católica Dom Bosco, Campo Grande, MS (license number 001/2017).

### 2.2 Sample collection and biometric data

For blood sample and biometric data collection, as well as to mark individuals with earrings, animas were anesthetized with a chemical association between Ketamine (20 mg/kg) and Xilazine (2 mg/kg) administered intramuscularly. The following data were recorded from each animal: capture point, sex, weight, tail and total length. The body condition was calculated by standardized residuals from an ordinary linear regression between body mass (g) and total length (mm) of individuals, while accounting for age and sex effects (Santos et al., 2018).

Once the animal was sedated, a trichotomy was performed on the basal area of the tail, followed by asepsis with antibacterial soap, iodized alcohol and alcohol 70% (three times each one). Blood samples were obtained from the lateral caudal veins with the aid of hypodermic needles (13 × 0.3) and syringe (3 mL), conditioned in tubes with anticoagulant (EDTA) and kept under refrigeration until the laboratory analysis. The animals were released at the capture point after recovery of anesthesia.

### 2.3 Laboratory procedures

The health of opossum was inferred, mainly, by means of blood parameters. Packed cell volume (PCV), red blood cell counts (RBC), and white blood cell counts (WBC) were measured up to 8 h after blood collection in Neubauer chambers, as described by Voigt (2000). Mean corpuscular volume (MCV) was calculated based on the RBC and PCV values. Leukocyte (eosinophils, lymphocytes, monocytes, and neutrophils) counts were performed using blood smears fixed with methanol and stained with Giemsa (Parreira et al., 2016).

Molecular detection of *T. cruzi* infection was performed by nested polymerase chain reaction (nPCR). Genomic DNA was extracted from 200 μL of blood with EDTA using the QIAamp Blood DNA Mini Kit (Qiagen) according to the manufacturer's instructions. Total DNA was diluted with 50 μL elution buffer and stored at −20 °C until molecular diagnosis. A variable region of the trypanosome 18S rRNA gene (600 bp), with external primers TRY927F and TRY927R, and internal primers SSU561F and SSU561R was used as a target, according to Smith et al. (2008). Furthermore, positive samples in the 18S rRNA had the primers D71 and D72 used to amplify a conserved sequence of the large subunit of the ribosomal DNA gene (24Sα rDNA) in *T. cruzi*, according to Souto and Zingales (1993). Each reaction included sterile distilled water instead of DNA as negative control, and positive control samples from *T. cruzi* strains. PCR products were visualized in 2% agarose gel after ethidium bromide staining under ultraviolet light.

### 2.4 Statistical analysis

Shapiro-Wilk test was performed to verify the normality of the hematological data. Subsequently, paired-t-test or paired-Wilcoxon test were used to compare differences in means between males and females. The simple linear regression was used to determine the relationship between the body condition and the parasitism by *T. cruzi* with the hematological data. We evaluated the health condition of *D. albiventris* in terms of: (a) PCV, RBC, and MCV as anemia indicators; (b) monocyte and neutrophil counts as indicators of infection responses; and (c) lymphocyte counts as indicators of immune investment. Additionally, we determined the direct and indirect influences of infection by *T. cruzi* in relation to anemia, infection responses, immune investment and body condition through path analysis following Santos et al. (2018). The critical level of significance was p < 0.05. All analyzes were performed in the R software (R Development Core Team, 2015).

## 3 Results

We sampled 40 individuals of *D. albiventris*, 26 males and 14 females. The molecular diagnosis showed that 32.5% (13/40) of the opossums were infected by *T. cruzi*. The statistical analysis revealed that individuals parasitized by *T. cruzi* showed lymphocytosis (3.4 ± 1.5) and eosinophilia (0.09 ± 0.13) when compared with non-infected animals (2.3 ± 1.3 and 0.03 ± 0.05), respectively (Table 1). No significant difference was observed between the averages of hematological variables in relation to gender and body condition.

**Table 1:** Mean and standard deviation of hematological values for *Didelphis albiventris*, infected and non-infected by *Trypanosoma cruzi* (Tc), captured in fragments of Campo Grande, Mato Grosso do Sul, Brazil.

*Didelphis albiventris*	Non infected (n = 27)	Tc positive (n = 13)
RBC (x10^6^)	2.7 ± 0.9^a^	3.2 ± 0.9^a^
PCV (%)	34.7 ± 5.2^a^	35.8 ± 3.9^a^
MCV (fl)	145 ± 59^a^	113 ± 21^a^
WBC (x10^3^)	4.9 ± 1.9^a^	5.7 ± 1.9^a^
Neutrophils (x10^3^)	2.4 ± 1.1^a^	2.2 ± 1.0^a^
Lymphocytes (x10^3^)	2.3 ± 1.3^a^	3.4 ± 1.4^b^
Monocytes (x10^3^)	0.08 ± 0.09^a^	0.08 ± 0.06^a^
Eosinophils (x10^3^)	0.03 ± 0.05^a^	0.09 ± 0.13^b^

Different letters represent statistical difference (p < 0.05).

**Fig. 1:**
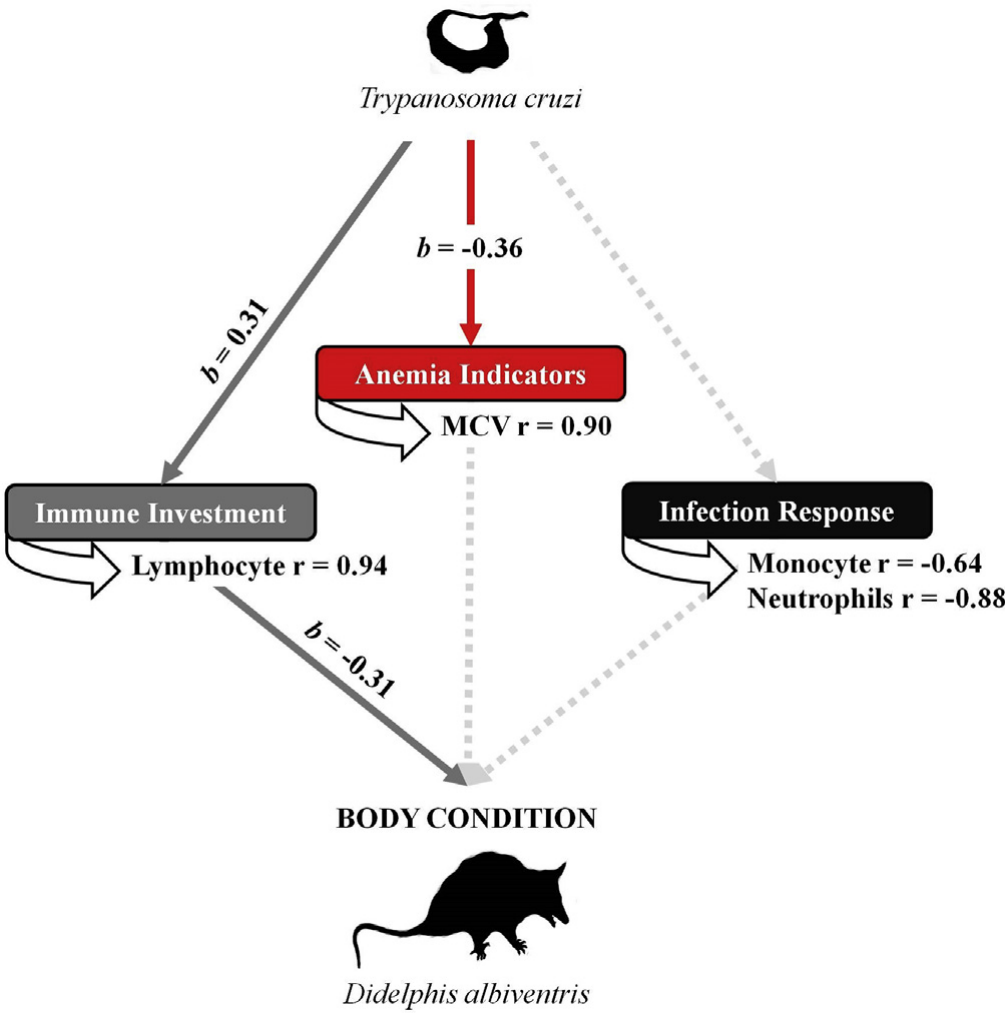
Path analysis showing direct and indirect influences of infection by *Trypanosoma cruzi* in relation to anemia, infection responses, immune investment and body condition of *Didelphis albiventris*.

Path analysis showed that *T. cruzi* (path coefficient = 0.31, p < 0.05) resulted in increased numbers of lymphocytes (r = 0.94) and indirectly decreased the body condition (path coefficient = - 0.31, p < 0.05) of opossums. Moreover *T. cruzi* infection resulted in a direct effect (path coefficient = −0.36, p < 0.05) on the decrease of MCV (r = 0.90) when compared with non-infected opossums, however with no effects on body condition (Fig. 1).

## 4 Discussion

The hematological parameters of free-living *Didelphis* spp. must be interpreted carefully since factors such as parasitism, age, season, feeding conditions and differences between species of *Didelphis* have been recorded (Casagrande et al., 2009; Carneiro et al., 2010; Tarragona et al., 2011). Our results showed significant difference for the hematological values between infected and non-infected opossoms, concerning the averages of lymphocytes and eosinophils. Moreover, no significant difference was observed between genders, which is in agreement with Casagrande et al. (2009). However, Tarragona et al. (2011) found significantly higher neutrophils values for females of *D. albiventris*.

The low RBC values recorded in our study were also documented in females of free-living *Didelphis aurita* (Casagrande et al., 2009), and may be associated to different infections or adaptive syndromes. In fact, parasites that lyse red blood cells and the consequently decrease RBC values, such *Babesia* spp. and haemotropic bacteria, reported in *Didelphis* spp. (Herrera; Urdaneta-Morales, 1991; Messick et al., 2002; Soares et al., 2017) may be the cause of reductions in erythrocyte values found in the present study. Moreover, hepatic lesions recorded in *Didelphis* sp. infected by *T. cruzi* (Carnevali et al., 2017) may compromise the production of erythropoietin, a glycoprotein that regulates the proliferation and differentiation of hematopoietic progenitor cells in the bone marrow (Naito et al., 2015).

Concerning adaptative disorders, the observed low RBC values in the opossums may be associated to a deficiency of some nutritional elements necessary to erythrocytes production such as vitamin B, folic acid and iron. Furthermore, the lower RBC with high MCV (>100) values recorded in the sampled animals indicated a macrocytic anemia, often caused by vitamin B12 deficiency and/or folic acid scarcity, commonly found in animal protein and leafy greens vegetables, respectively (Ribeiro, 2005; Hughes et al., 2013; Gonçalves, 2018). In this sense, it is possible that the forest fragments of Campo Grande were not adequately supplementing the animals.

The lower MCV values recorded in *T*. *cruzi*-infected opossums, compared with non-infected animals, could be correlated to deficient hemoglobin synthesis due to hypoferremia (Massey, 1992; Krishnamurthy et al., 2007; Oates, 2007). In fact, the multiplication of intracellular amastigote forms of *T*. *cruzi* is strongly associated to the influx of iron into the cell (Lalonde; Holbein, 1984; Loo; Lalonde, 1984). The lower MCV values was also reported in free-living coati (*Nasua nasua*) naturally infected by *T. cruzi* in the Pantanal biome by Santos et al. (2018).

Lymphocytosis, found in our study in infected animals, may be associated with a potent stimulation of cellular and humoral immune response, characteristic of chronic phase of *T. cruzi* infection in humans and dogs (Barr et al., 1991; Cordeiro et al., 2001; Duz et al., 2014; Girones; Fresno, 2003). However, Jansen et al. (1991) reported that IgG antibodies are unable to prevent the chronic phase in *Didelphis marsupialis* experimentally infected. Moreover, it has been reported that lymphocytes act together with neutrophils and monocytes to repair the tissue damage caused by *T*. *cruzi* amastigote forms (Luna-Gomes et al., 2014). Besides that, Guedes et al. (2012) observed that lymphocytosis was associated in dogs experimentally infected by *T. cruzi*.

The indirect effect (immune investment) of *T. cruzi* infection on the opossum's body condition observed in our study can be associated with a potent stimulation of humoral immune response, characteristic of trypanosome infections (Cordeiro et al., 2001; Soares et al., 2001; Girones et al., 2003; Gutierrez et al., 2009). Therefore, the strong production of immune complexes causes severe pathologies in the different tissues, widely reported in trypanosome infections (Barret-Connor et al., 1973; Pattison; Steward, 1985). In fact, severe tissue changes in both natural and experimental *T. cruzi* infections in *D. virginiana* and *D. marsupialis* respectively have been observed (Araujo et al., 1996; Carnevali et al., 2017). The negative influence of *T. cruzi* on body condition through immune investment was also observed in infected wild carnivore *Nasua nasua* in the Pantanal of Mato Grosso do Sul (Santos et al., 2018).

The increase of eosinophils observed in *T. cruzi* infected opossums has been largely recorded in human patients with Chagas Disease (Koberle, 1968; Nogueira et al., 1982; Villalta and Kierszenbaum, 1984; Kierszenbaum et al., 1986), related to the occurrence of fibrosis in the extracellular matrix (Noguchi et al., 1992; Lopes and Chapadeiro, 1997; Oliveira, 2013). In fact, neutrophils and eosinophils have been implicated in killing epimastigotes and trypomastigotes of *T. cruzi* in an antibody dependent cellular cytotoxicity-type mediated mechanism (Sanderson et al., 1977; Kierszenbaum, 1979; Madeira et al., 1979; Olabuenaga et al., 1979; Sanderson; de Souza, 1979; Kipnis et al., 1981). Moreover, eosinophils are also present in the bone marrow and peritoneal cavity of *T.* cruzi-infected mice (Rowland et al., 1983). Moreover, the eosinophilia observed in the *T. cruzi* infected opossums can be the result of co-parasitism by helminths, as already observed by Monteiro et al. (2010) in *Leontopithecus* spp naturally parasited.

## 5 Conclusion

Overall, our results suggest that *T. cruzi* infection may pose a risk to health of opossums since lymphocytosis may cause a secondary damage on body condition of infected animals. Moreover, due to an ancient relationship between *T. cruzi* and *D. albiventris*, this marsupial species may be an important reservoir host in the maintenance of this multi-host protozoan in the urban area of Campo Grande city.

## Declarations of interest

None.
